# Shoot or Don’t Shoot? Tactical Gaze Control and Visual Attention Training Improves Police Cadets’ Decision-Making Performance in Live-Fire Scenarios

**DOI:** 10.3389/fpsyg.2022.798766

**Published:** 2022-02-23

**Authors:** Benedikt Heusler, Christine Sutter

**Affiliations:** Institute of Traffic and Engineering Psychology, German Police University, Münster, Germany

**Keywords:** police, law enforcement, gaze control, training, visual attention, decision-making

## Abstract

Police officers often encounter potentially dangerous situations in which they strongly rely on their ability to identify threats quickly and react accordingly. Previous studies have shown that practical experience and targeted training significantly improve threat detection time and decision-making performance in law enforcement situations. We applied 90-min traditional firearms training as a control condition (35 participants) and a specifically developed intervention training (25 participants) to police cadets. The intervention training contained theoretical and practical training on tactical gaze control, situational awareness, and visual attention, while the control training focused on precision and speed. In a pre- and posttest, we measured decision-making performance as well as (tactical) response preparation and execution to evaluate the training. Concerning cognitive performance training (i.e., decision-making), the number of correct decisions increased from pre- to posttest. In shoot scenarios, correct decisions improved significantly more in the intervention group than in the control group. In don’t-shoot scenarios, there were no considerable differences. Concerning the training of response preparation and execution in shoot scenarios, the intervention group’s response time (time until participants first shot at an armed attacker), but not hit time, decreased significantly from pre- to posttest. The control group was significantly faster than the intervention group, with their response and hit time remaining constant across pre- and posttest. Concerning the training of tactical action control, the intervention group performed significantly better than the control group. Moreover, the intervention group improved the tactical handling of muzzle position significantly. The results indicate that a single 90-min session of targeted gaze control and visual attention training improves decision-making performance, response time, and tactical handling of muzzle position in shoot scenarios. However, these faster response times do not necessarily translate to faster hit times – presumably due to the motor complexity of hitting an armed attacker with live ammunition. We conclude that theory-based training on tactical gaze control and visual attention has a higher impact on police officers’ decision-making performance than traditional firearms training. Therefore, we recommend law enforcement agencies include perception-based shoot/don’t-shoot exercises in training and regular tests for officers’ annual firearm requalification.

## Introduction

Law enforcement plays a vital role in providing communities with general security, preventing crimes, and detaining suspects. Although police officers often face potentially dangerous situations on duty, lethal encounters are fortunately isolated. However, attacks aimed at police officers’ lives can still happen at any time and without apparent indicators that allow officers to prepare for an escalating situation. Therefore, law enforcement personnel often find themselves in a challenging position between pursuing a community-oriented policing approach while also knowing that they may have to face deadly confrontations in an instant. Patrol officers, especially, are expected to manage the balancing act of being approachable helpers to their community and highly specialized tactical officers at the same time. To be prepared, police officers must rely on their situational awareness, ability to assess threats, and ability to react under stress (Helsen and Starkes, 1999; Vickers and Lewinski, 2012; Martaindale, 2021).

Situations in which a police officer shoots a citizen usually attract considerable public attention – especially if the citizen was unarmed. Therefore, it should be in everyone’s interest to identify and implement measures to avoid these incidents and reduce harm on both sides. One important factor that negatively influences police officers’ performance in use-of-force situations is stress (Nieuwenhuys et al., 2009, 2012; Akinola and Mendes, 2012). High levels of anxiety critically reduce perception capability and situational awareness. Biggs et al. (2021) showed that military personnel, who undergo stress-inoculation training, are less likely to shoot at an unarmed person. Another aspect worth mentioning is tactical considerations. Taylor (2020) demonstrated in an experimental setting with a firearms simulator that police officers can reduce the risk of mistakenly shooting an unarmed suspect without sacrificing a considerable amount of time by taking a lower muzzle position.

Police officers’ and military personnel’s primary source of information is visual perception – especially when identifying objects and assessing threats. With more than one-third of the human brain being affiliated with visual perception, this primary system of sensory information processing far outweighs other senses in potentially lethal law enforcement situations (Findlay and Gilchrist, 2003; Alt and Darken, 2008; Sutter and Ladwig, 2012; Ladwig et al., 2013; Körber, 2016; Heusler and Sutter, 2020a). Studies showed that law enforcement expertise and training facilitate performance in visual search tasks related to potential threats (Körber et al., 2007; Vickers and Lewinski, 2012; Martaindale, 2021). Moreover, Körber (2016) showed that visual priming can positively influence the identification and visual search of dangerous objects and weapons.

However, human visual perception has its limitations on multiple levels. For one, there are physical limitations, like the narrow sharp corridor within our field of vision (foveal vision; about 2°) and the fact that new visual information cannot be processed between fixations (Yarbus, 1967; Irwin, 1992; Hoffman, 1998; Salvucci and Goldberg, 2000; Castelhano et al., 2009; Carrasco, 2011). Another crucial aspect in this context is attention. Although visual information may be obtained and processed by the visual system, further cognitive processing depends on stimulus salience (i.e., prominence of stimulus features to attract attention) and locus of attention (i.e., where attention is focused). Vice versa, attention can actively influence gaze and visual perception (Groner and Groner, 1989; Findlay and Gilchrist, 2003; Henderson et al., 2007; Carrasco, 2011). Therefore, visual perception is not merely passive but an active process, which Findlay and Gilchrist (2003) described as “active vision.”

These physical limitations, a lack of attention, anxiety, inexperience, and other factors may seriously hamper threat assessment in law enforcement situations. Taking this into consideration, it becomes apparent how important awareness and practical training are (Ho, 1994; Dewhurst and Crundall, 2008; Nieuwenhuys and Oudejans, 2010, 2011; Nieuwenhuys et al., 2012; Biggs et al., 2015, 2021; Donner et al., 2017; Donner and Popovich, 2019). Therefore, it is essential to teach law enforcement officers what to expect, what to focus on (both gaze and attention), and what factors to base shoot decisions on.

Amini and Vaezmousavi (2020) found that an external-relevant attentional focus improved the performance of elite military shooters. They claim that this attentional focus strategy was more effective in this context than other attentional focus strategies. Hamilton et al. (2019) showed that cognitive training can improve shooting performance in law enforcement situations. These findings are consistent with Preddy et al. (2019), who suggested that cognitive readiness in the context of critical encounters in a law enforcement context should be supported by skill training in the areas of domain and prerequisite knowledge, pattern recognition, and situational awareness.

One of police officers’ hardest decisions is whether to shoot and risk shooting an unarmed person or to hold fire and risk being killed by an armed person. This dilemma is already challenging enough; however, officers might have to make such decisions in a split second and while under extraordinary levels of anxiety. Additionally, environmental circumstances (e.g., dim light or distractions) can make it hard or even impossible for an officer to detect a deadly threat and react before it is too late. Kruke and Henriksen (2020) found that Norwegian police officers showed a tendency to hold their fire in real confrontations until life-threatening situations materialized into actual attacks – resulting in potentially avoidable, imminent danger. In an experiment, Blair et al. (2011) showed that even under near-perfect conditions, it may not be possible for police officers to shoot at an armed attacker in time. It took suspects, pointing a gun to the ground, on average 360 ms to raise the firearms, aim, and shoot at a police officer. Even though the officers started with their guns already aimed at the attacker, they still needed 380 ms after the suspect’s initial movement to return fire (not necessarily hitting the attacker). These response times reveal the importance for law enforcement personnel to anticipate potential attacks and identify threats quickly. Suss and Raushel (2019) experimented using video scenarios in which actors either pulled a revolver or a wallet. Participants were relatively unbiased in their anticipation during the first part of the drawing motion. However, they tended to anticipate a weapon more frequently than a non-weapon as more of the draw motion was revealed.

The decision to shoot or not to shoot must be made consciously – even under elevated anxiety levels. Simply being frightened or surprised by a complex situation is not a legitimate reason to shoot when the decision is not based on a valid threat assessment. Vice versa, being overwhelmed and choking under pressure should not be the reason for not shooting. Biggs and Pettijohn (2021) showed in realistic military scenarios that inhibitory control plays a considerable role in shoot/don’t-shoot decision-making.

The goal of training police officers must be to increase the probability of correct decision-making in real-world situations by training under comparable conditions. Moreover, handgun qualification tests for police officers should involve more than marksmanship elements. Law enforcement situations are complex and unpredictable and cannot be simulated by merely having officers shoot at predefined, static targets (Morrison and Vila, 1998; Helsen and Starkes, 1999).

Most previous studies examining law enforcement personnel’s shoot/don’t-shoot performance (e.g., Akinola and Mendes, 2012; Nieuwenhuys et al., 2012; Davies, 2015) used non-lethal training equipment, response boxes, or static targets to measure decisions and performance. As a result, empirically sound data of police officers reacting to dynamic video scenarios using live ammunition in experimental settings are scarce.

Based on the results of previous studies and the current theoretical framework, we designed an intervention training focusing on the following key elements.

• Realism (using pictograms and photographs as targets instead of abstract geometrical shapes);
• Situational awareness (raising awareness toward the need to assess threat-levels, e.g., “are the suspect’s hands visible?”);
• Tactical gaze control (training participants to actively shift their gaze on tactically crucial regions, like a suspect’s hand- and hip region); and
• Visual attention (training participants to be vigilant toward critical visual stimuli, e.g., weapons).

We expected this targeted training to improve police officers’ shoot/don’t-shoot performance. We also expected this type of training to raise officers’ awareness of the importance of visual perception, causing them to keep their eyes open longer and raise their weapons later into their line of sight. Therefore, we formulated the following six hypotheses.

Compared to police cadets who receive active control training, we hypothesized that police cadets who complete 90-min firearms training on tactical gaze control and visual attention improve their performance from pre- to posttest by

• More often making the correct decision to shoot in shoot scenarios (hypothesis 1);
• More often making the correct decision not to shoot in don’t-shoot scenarios (hypothesis 2);
• Shooting faster in shoot scenarios (hypothesis 3);
• Hitting the attacker faster in shoot scenarios (hypothesis 4);
• Bringing their gun later up to eyesight level before shooting (hypothesis 5); and
• Keeping their eyes open longer right before shooting (hypothesis 6).

## Materials and Methods

The personnel responsible for conducting the experiment consisted of three people. The main instructor and the supporting instructor (the primary author of the current study) were in charge of operating the firing range, ensuring compliance to safety regulations at all times, and providing the training. Both instructors were experienced police trainers and licensed firearms instructors. In addition, a research assistant oversaw the timing of sequences, supervised the participants, and supported setting up the pre- and posttest material.

### Participants

A total of 95 police cadets volunteered for the study. All participants were students of the 3-year bachelor’s degree course “Polizeivollzugsdienst – Schutzpolizei” (Law Enforcement – Uniformed Police) at a German State Police Academy. All participants were in their third semester and had already received basic firearms training and lectures in fundamental police tactics. The study’s training was applied to eight classes with about 12 persons each. Although the study was part of the regular (and mandatory) firearms training, the students were not obliged to participate in the experimental part of the training. However, all cadets volunteered and gave their written consent after being handed a comprehensive information sheet. The study was reviewed and approved by the German Police University’s ethical review committee and the University of Applied Sciences for Police and Administration. Except for the participants’ genders, no personal data were gathered for the study. The results were stored anonymously using random three-digit identification numbers assigned to the participants.

We randomly assigned four classes (49 participants) to the intervention training and four classes (46 participants) to the control training. All participants received either the intervention or control training. Due to high occupancy of the firing range and the resulting time limitations, only a random 69 out of the 95 participants could be considered for both the pre- and posttest. Even though all of these 69 participants completed both test parts, technical issues resulted in improper data recording, so that the posttest data of nine participants were not available. This left us with a total of 60 participants (*N* = 60; 15 female; 45 male), whose pre- and posttest data were evaluable. Table 1 provides the group characteristics. The intervention group consisted of 35 participants (10 female; 25 male), while the control group consisted of 25 participants (5 female; 20 male).

**TABLE 1:** Group characteristics.

*N* = 60	Intervention group	Control group
Number of participants	35	25
Gender (female/male)	10/25	5/20

Since we created a new research design for the current study, we could not use a power analysis to estimate the necessary sample size. However, we oriented ourselves by comparable studies with between-subject designs (e.g., Nieuwenhuys et al., 2012; Vickers and Lewinski, 2012), that investigated 24–36 participants with 11–18 participants per group. Considering that these studies generated effect sizes up to 0.77 and 1.52, we expected our sample size to be sufficient.

### Pre- and Posttest

**FIGURE 1:**
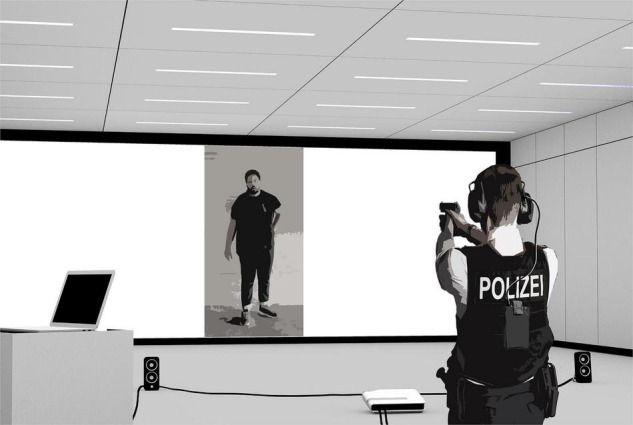
The experimental setup of the pre- and posttest.

The pre- and posttests were conducted in an indoor firing range (with an integrated digital target-projection system) of a German State Police Academy. The range’s target canvas consisted of two backlit layers of continuous paper sheets that could be moved in opposite directions to reset the visible hits. Figure 1 depicts the experimental setup for the pre- and posttest. The participants stood on a marked spot 6 m from the paper target canvas. A mobile projector (Casio XJ-A255V) and two stereo audio speakers (Logitech Z200) were placed on the floor between the participant and the canvas. The speakers and the projector were connected to a laptop computer that controlled the stimuli presentation. The firing range was dimly lit to allow for better visibility of the projected targets on the canvas.

The participants used a Heckler & Koch P30 V2 handgun, their standard-issue 9 mm service pistol, and were given a choice of two grip sizes. The pistol was loaded with a magazine containing 15 rounds of live ammunition. For the recording of eyelid movements and first-person videos, participants wore the “Pupil Invisible” glasses by Pupil Labs – a lightweight (46.9 g) mobile device with individual eye cameras (resolution: 192 × 192 pixels) and a front camera (resolution: 1,088 × 1,080 pixels; field of vision: 82°) attached to the left temple. The Pupil Invisible glasses also served as eye protection and substituted the regular shooting safety glasses. In addition, all participants wore earmuffs (3M Peltor) and earplugs (Bilsom 303L) for hearing protection. The double hearing protection was introduced because the temple tips of the Pupil Invisible glasses were slightly thicker than the temple tips of regular shooting safety glasses and we could not entirely rule out that this might have reduced the effectiveness of the earmuffs. The Pupil Invisible glasses were connected to a OnePlus 6 mobile phone in a radio pouch attached to the back of the participants’ standard issue ballistic vests. The two experimenters, both licensed firearms instructors, wore standard-issue ballistic vests and earmuffs like the participants.

**FIGURE 2:**
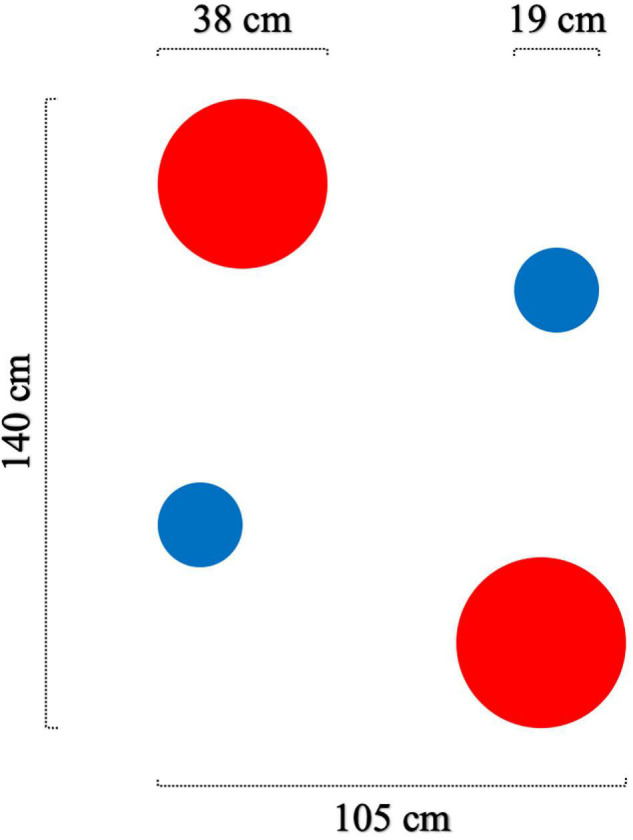
Target arrangement for task 1.

Tasks and stimuli were the same for pre- and posttest. The dependent variables for each task are described in the section Design and Statistical Analyses. Task 1 served as a manipulation check to see whether the two types of training affect “traditional” firearms proficiency and improve the participants’ ability to cope with the speed–accuracy tradeoff. The goal was to hit as many static targets as possible in the least amount of time. Figure 2 depicts the stimuli for task 1: the targets were two red circles (ø 38 cm) and two blue circles (ø 19 cm). Participants were instructed to first shoot at two red circles and then at two smaller blue circles as accurately and fast as possible. They had to fire at each circle once and then move on to the next one – even if they missed. This task resembles the most common tests used by German law enforcement agencies for annual firearm requalification.

Task 2 tested the participants’ shoot/don’t-shoot performance in video scenarios. At the outset, a briefing sheet provided the background story to the video scenarios. Participants were informed that they were on patrol duty when they identified a wanted fugitive. This male fugitive was a suspect in an armed robbery of a jewelry store that had taken place a couple of hours earlier. They were also informed that the fugitive was most likely armed and unpredictable. Next, the information sheet provided current mug shots for the participants to recognize the fugitive in the scenarios. Furthermore, the participants were instructed that they would have to complete multiple video scenarios that were all based on the background story. Every participant approached each scenario as a single officer; no backup officers were present. They were instructed to deal with the situation as realistically as possible, including talking to the fugitive. Tactical choices, like verbal communication or the muzzle position of their service pistol, were up to the participants. The dependent variables for each task are described in the section Design and Statistical Analyses.

**FIGURE 3:**
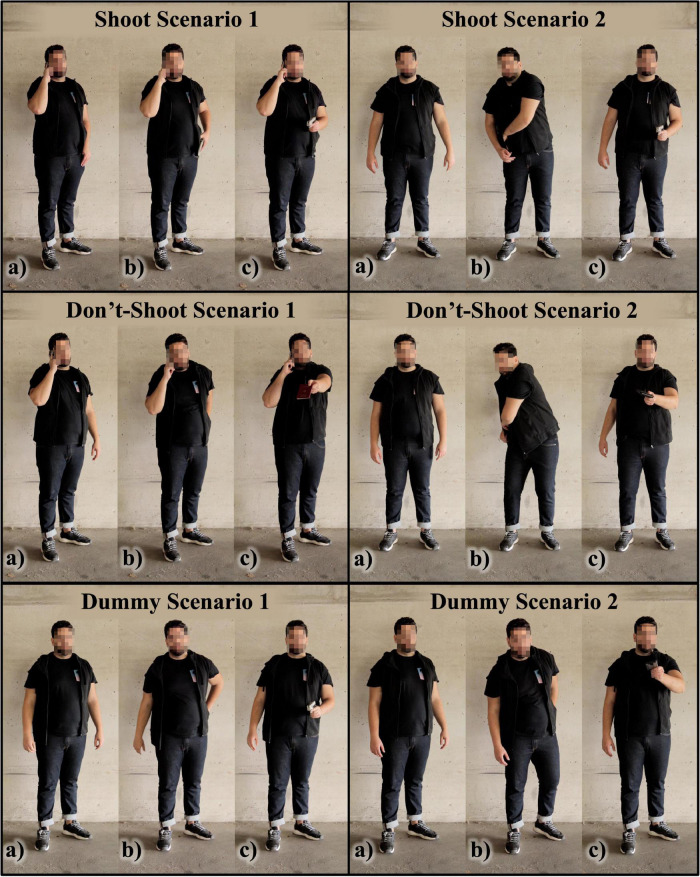
The six different scenarios of task 2. **(a)** The initial situation, **(b)** the drawing motion, **(c)** the suspect’s final position with the drawn object.

Figure 3 depicts the six experimental scenarios (the actor’s face is blurred for publication but was visible to the participants during the study). Task 2 included two shoot scenarios (Figure 3, upper), two don’t-shoot scenarios (Figure 3, middle), and two dummy scenarios (Figure 3, lower). The video scenarios always showed the same male suspect from the instruction sheet in front of a neutral concrete wall. In the shoot scenarios, the suspect drew a gun and pointed it at the participant. In the don’t-shoot scenarios, the suspect drew a harmless object (e.g., passport) and pointed it at the participant. The pre- and posttest each comprised three scenarios. The first and second scenario were either shoot and don’t-shoot, or don’t-shoot and shoot, respectively. The third scenario was always a dummy scenario (i.e., either a shoot or a don’t-shoot scenario, selected at random). The inclusion of the dummy scenario was designed to prevent participants from knowing exactly how many shoot scenarios and how many don’t-shoot scenarios they would encounter within each three-scenario test. Therefore, within each test, participants could encounter one shoot scenario and two don’t-shoot scenarios, or two shoot scenarios and one don’t-shoot scenario—in any order. The scenarios were comparable regarding the suspect’s drawing speed and general behavior.

In shoot scenario 1 (S1) and don’t-shoot scenario 1 (D1), the suspect held a mobile phone to his ear with his right hand throughout the scenario to simulate a phone conversation. His left hand remained at waist level until he drew an object after about 13 s and pointed it toward the camera. In S1, the object was a silver pistol and in D1 a red passport.

In shoot scenario 2 (S2) and don’t-shoot scenario 2 (D2), the suspect’s hands were initially empty and visible. After about 10 s, he reached diagonally into his jacket’s inside pocket, drew an object with his left hand, and pointed it toward the camera. In S2, the object was a silver pistol and in D2 a wallet.

In dummy scenario 1 and dummy scenario 2, the suspect’s hands were initially empty and visible. He then reached behind his back with his left hand, drew an object from his back pocket, and pointed it toward the camera. In dummy scenario 1, the object was a silver pistol and in dummy scenario 2 a mobile phone.

The scenarios were designed in close cooperation with experienced police trainers to ensure realism and tactical unambiguity. The shoot scenarios left no room for vagueness or hesitation, as the suspect posed an immediate threat to the participant’s life by drawing a pistol and aiming it at them. Therefore, participants had to shoot their service pistol at the suspect in the shoot scenarios. On the other hand, in the don’t-shoot scenarios, shooting at the unarmed suspect was an apparent mistake. For video production, a single-lens reflex camera (Canon EOS 550D) on a tripod was used. We set up the camera approximating its position to human eyesight level (165 cm above ground) and filmed from the same distance (6 m) that the participants later stood in front of the canvas. This allowed us to project the videos in life-size (suspect’s height: approximately 180 cm) while also simulating a natural visual perspective. The suspect’s dark clothes contrasted sharply with the light-colored background. This allowed participants to see the subject’s movements and the objects clearly.

### Training

As described above, we assigned eight classes to receive one of the two types of training. Both the control and intervention training were conducted in groups, lasted 90 min, and focused on visual perception. Although both forms of training included elements of visual perception and decision making, the theory-based intervention training focused on teaching the aforementioned aspects of attention and tactical gaze control. Each participant fired 30 live rounds and stood 8 m from the target canvas, regardless of the type of training they received. All exercises in the training were static and did not include any video scenarios, ensuring comparability by not favoring any group in preparation for the posttest. Table 2 provides a comparison of both training concepts.

**TABLE 2:** Comparison of both training concepts.

	Intervention training	Control training
Context	● Realism	● Abstract visual stimuli
Content	● Situational awareness ● Tactical gaze control ● Visual attention	● Precision ● Speed
Training goals	● Participants’ increase shoot/don’t-shoot decision-making proficiency ● Participants keep both eyes open while using their service pistol ● Participants maintain a low muzzle position longer before firing	● Participants increase their proficiency in dealing with the speed–accuracy tradeoff ● Participants decrease response time after a visual “go” signal
Theoretical input (30 min)	● Safety protocol ● Basics of human visual perception ● Theoretical introduction to tactical gaze control (where to look) ● Importance of attention (what to be vigilant about) ● Situational awareness (what to expect)	● Safety protocol ● Theoretical introduction to the speed–accuracy tradeoff ● Proper use of the pistol’s sights for maximum accuracy ● Proper movement of the pistol to ensure maximum speed
Practical exercises (60 min)	● Exercise 1 (warm-up): Six rounds on a torso-sized rectangle. ● Exercise 2: Six rounds on circles after small visual impulse. First introduction to visual target recognition. ● Exercise 3: Nine rounds on indicated human silhouettes with pictogram hands holding various objects. First introduction to threat-based shoot/don’t-shoot decisions. Participants are encouraged to focus gaze and attention on the hands and give verbal orders. ● Exercise 4: Nine rounds on life-sized photographs of persons in various situations holding different objects. Same objective as in exercise 3 but increased realism.	● Exercise 1 (warm-up): Five rounds on squares of different sizes. ● Exercise 2: Four rounds on colored circles with a finishing round on a smaller circle. Participants were encouraged to focus on the correct use of their gun’s sights. ● Exercise 3: Ten rounds on shrinking squares. Practical introduction to the effects of the speed–accuracy tradeoff. ● Exercise 4: Five rounds on the same circles as in exercise 2. This time the order of the targets is indicated by their color. First introduction to visual target recognition. ● Exercise 5: Five rounds on a static bullseye target. Participants were encouraged to find their “sweet spot” between firing as fast and accurately as possible.

The training took place in the same indoor firing range as the pre- and posttest. The weapons and safety equipment were the same as in the pre- and posttest except that the participants wore standard-issue shooting safety glasses and earmuffs without additional earplugs. We used the firing ranges’ integrated ceiling-mounted projectors and computer to project the targets. All participants were given direct feedback on their performance (e.g., correct/wrong decisions, weapon handling, and shooting technique) after each practical exercise.

#### Control Training

The active control training resembled traditional law enforcement firearms training, in which the focus is set on identifying and hitting targets as fast as possible. These training goals match the requirements of the regular tests that police officers usually have to pass during annual firearm requalification. Additionally, the training aimed to improve the participants’ visual perception and decision-making performance in abstract and static shooting exercises.

The theoretical part of the control training focused on educating the participants about the speed–accuracy tradeoff and the most effective ways to ensure maximum precision in the least amount of response time possible. Thus, the instruction can be described as a conventional approach toward theoretical police firearms tactics.

In the practical part of the control training, the participants performed shooting exercises that gradually increased in complexity and difficulty. The targets were initially large and predefined but later shrunk in size and had to be identified by the participants. Some targets had to be shot in a given order while others had to be avoided, thus forcing the participants to actively search for the targets, identify them and make conscious decisions on whether to shoot or not. Even though the exercises increased in complexity, and decision-making tasks (based on shapes, numbers, and colors) became increasingly challenging, the participants were not confronted with threat-based decisions.

#### Intervention Training

The theory-based intervention training was newly designed and focused on tactical gaze control, attention, and situational awareness for detecting weapons on a suspect. It aimed at improving visual perception strategies and decision-making performance in realistic law enforcement scenarios.

In the theoretical part of the intervention training, participants were instructed to focus both gaze and visual attention on tactically crucial regions. For example, although a suspect’s face is very salient and may be a good indicator of their emotional state in most cases, it does not necessarily predict an upcoming attack or the actual danger level that a person poses. Therefore, police officers have higher chances of detecting and correctly identifying drawn objects by focusing both gaze and attention on a suspect’s hands and hip region (Heusler and Sutter, 2020a,b).

Furthermore, the theoretical intervention training taught the participants about situational awareness as a crucial factor in reducing risk in potentially lethal situations. Situational awareness in this context describes the ability to distinguish routine situations from situations with elevated risk potential. Nonetheless, situational awareness also encompasses the understanding that even seemingly harmless law enforcement situations can escalate. Thus, a vital element of the intervention training was to teach participants about the potential dangers of direct interaction with suspects while also sensitizing them to the possible adverse effects of being too expectant of attacks. Police officers can escalate situations unnecessarily if they solely focus on potential attacks rather than trying to de-escalate.

**FIGURE 4:**
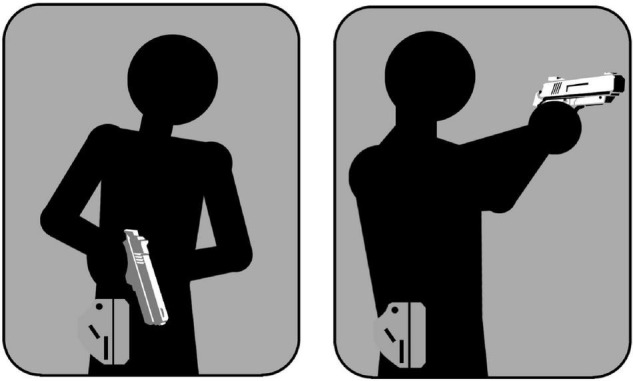
The compressed ready position **(left)** and the final shooting stance **(right)**.

The practical part of the training started with abstract targets (rectangles and circles), then progressed to semi-realistic targets (torso silhouettes with pictogram hands and objects) and ended with photographs of persons. As the targets became increasingly realistic with each new exercise, the participants were instructed to actively shift their attention and gaze toward the hands and hip region of the silhouettes and the photographs. Although the participants were not given instructions on which stance to take in any given situation, they were advised to take a compressed ready position with their service pistol’s muzzle pointing to the ground (Figure 4) when they assumed an elevated threat level. This advice was based on the previously described findings of Taylor (2020). The participants were also advised to keep both eyes open while shooting, ensuring maximum visibility of the suspect’s hands and hip region at all times.

Another aspect of the practical intervention training was to teach proper communication in law enforcement situations with elevated risk levels. Professional verbal communication is crucial for de-escalating situations, as is giving suspects clear orders before, during, and after an attack. The instructors demonstrated appropriate verbal communication tactics and reinforced the use of such tactics during the training. Although communication was neither documented nor analyzed in the study, it added to the workload and overall realism.

### Procedure

The procedure was the same for each class, regardless of whether they were assigned to the control group or the intervention group. We started by welcoming each class and introducing the research personnel. Then the participants were handed a comprehensive information sheet informing them about the study’s anonymous, voluntary, and confidential (information was not to be shared with fellow cadets) nature. Although the participants knew they were about to be part of an experiment, they were not yet told about the study’s focus on visual perception and decision-making. We only recorded and used the data of participants who gave their written consent after reading the information sheet. As all potential participants agreed to take part in the study, no data had to be excluded. The participants then did a 5-min written test on basic police firearms tactics, putting them in a tactical mindset without revealing the focus of the study. The participants did the subsequent pretest individually, taking about 4 min per person (8 min including the introduction).

Both the pre- and the posttest took about 60 min and required the participants to follow a predefined sequence of actions: one participant prepared for the upcoming practical shooting by reading the introductions for tasks 1 and 2, while another participant did the shooting at the range. During this time, the remainder of the class worked on a class assignment unrelated to the experiment. A research assistant oversaw the group exercise to ensure that no information about the study was exchanged between officers who had completed the experimental tasks and those who had not yet completed them.

In preparation for the upcoming practical shooting, participants first received a playing card, which they were asked to keep until the end of the experiment. The playing cards allowed us to match the participants to their corresponding data sets later without identifying them through personal data. Then, after reading the instructions for the shooting tests, the participants entered the shooting range and were equipped with hearing protection, the Pupil Invisible glasses, the mobile phone with the radio pouch, and a pistol with a fully loaded magazine (15 rounds). Participants had the choice between two different pistol grip sizes, depending on personal preferences.

All the instructions inside the shooting range were presented visually to ensure equal treatment of the participants. However, questions were answered if necessary. The presentations of the testing tasks 1 and 2 (for details, see section “Pre- and Posttest”) were preceded by a 3-s visual countdown to ensure that participants were ready. Before the scenarios in task 2, we presented a picture of the suspect to ensure that participants recognized him as the fugitive introduced in the instructions. The participants were also reminded that they could pick a stance and muzzle position of their choice.

To guarantee maximum variety and reduce confounding variables, we constructed 16 counterbalanced orders of the shoot and don’t-shoot scenarios (Table 3). Participants in each group were randomly assigned to one of these 16 orders.

**TABLE 3:** The 16 possible variations of scenario orders (S1/2 = shoot scenario; D1/2 = don’t-shoot scenario; SD/DD = shoot and don’t-shoot dummy).

Counterbalanced order	Pretest scenario order			Posttest scenario order		
	First	Second	Third	First	Second	Third
1	S1	D1	SD	S2	D2	DD
2	S1	D1	DD	D2	S2	SD
3	S1	D2	SD	D1	S2	DD
4	S1	D2	DD	S2	D1	SD
5	D1	S1	SD	S2	D2	DD
6	D1	S1	DD	D2	S2	SD
7	D1	S2	SD	S1	D2	DD
8	D1	S2	DD	D2	S1	SD
9	S2	D1	SD	S1	D2	DD
10	S2	D1	DD	D2	S1	SD
11	S2	D2	SD	S1	D1	DD
12	S2	D2	DD	D1	S1	SD
13	D2	S1	SD	D1	S2	DD
14	D2	S1	DD	S2	D1	SD
15	D2	S2	SD	S1	D1	DD
16	D2	S2	DD	D1	S1	SD

*The dummy scenarios served to prevent participants from recognizing patterns and were not included in the data analysis.*

After completing the pretest tasks, the participant returned the experimental equipment to the experimenters and joined the rest of the class to continue working on the class assignment. After sanitizing the equipment, the experimenters welcomed the next participant and proceeded with the following pretest.

The following 90 min consisted of the training, which comprised approximately 30 min of theory and 60 min of practical shooting exercises. The practical exercises were conducted simultaneously on two firing lanes with two firearms instructors, giving individual and instant feedback on the participants’ performances. While awaiting their turn to complete the practical exercises, participants were encouraged to work on weapon handling and run “dry” exercises at the rear of the shooting range.

The posttest was similar to the pretest and lasted another 60 min. Afterward, the participants tidied up the range and debriefed with the experimenters. During the debriefing, participants were encouraged to ask questions and give feedback.

In total, the experiment lasted about 4 h per group. Data were collected on 4 days (two groups per day) in October and November 2020.

### Design and Statistical Analyses

The current study followed a quasi-experimental, counterbalanced, controlled, repeated-measures design. We used the simplified term “experiment” to facilitate readability throughout the paper. The first measure took place in the form of a pretest before the intervention, while the second measure took place in the form of a posttest after the intervention. We used a control group, which received traditional training and an intervention group that received the new, specially designed training.

As described above, task 2 of the pre- and posttest comprised a shoot scenario and a don’t-shoot scenario (not necessarily in that order) with a dummy scenario at the end. The dummy scenario’s sole purpose was to prevent participants from recognizing patterns and anticipating upcoming tasks. Therefore, the dummy scenarios were not considered in the statistical analysis.

*Hit Factor:* The *Hit Factor* is a quotient commonly used in evaluating police officers’ shooting performance. In the present experiment, it serves as a manipulation check to evaluate participants’ performance in the traditional firearms exercise (task 1). The *Hit Factor* was calculated by dividing the number of hits (maximum one hit per target; maximum four hits total) by the time taken to complete the task. It describes the participant’s performance in task 1 based on accuracy and time (from the start of the exercise until the last shot). The higher the *Hit Factor*, the better the participant’s performance. Note that the quotient could exceed 1.0 if participants completed the task in less than 4 s. One control group participant was excluded from the analysis since they did not follow the instructions and shot twice at every circle instead of once (both in the pre- and posttest).

For the statistical analysis, we first calculated a repeated-measures ANOVA with the between-subject factor Group and the within-subject factor Time. We then calculated separate repeated-measures ANOVAs for each group to investigate their respective pre- to posttest development.

*Decisions:* This variable aimed to investigate the two hypotheses on decision-making and was documented separately for the shoot (hypothesis 1) and don’t-shoot scenarios (hypothesis 2). It describes the participants’ decision progress from pre- to posttest.

When a participant shot in a don’t-shoot scenario or before the drawn object was first identifiable, it was automatically considered a wrong decision (false positive). When a participant did not shoot at an armed attacker after the drawn pistol had been identifiable for 2 s, the decision was also automatically considered wrong (i.e., false negative or miss). We set 2 s as the maximum response time in consultation with active police trainers and firearms experts. After 2 s elapsed following the presentation of a clear threat, it is safe to assume that the officer either failed to detect the threat or mistakenly decided not to shoot despite the threat. Two seconds are sufficient for an armed attacker to fire multiple deadly shots at an officer in a real-life scenario. Correct decisions will be referred to as “correct positive” (participant shot in a shoot scenario) and “correct negative” (participant did not shoot in a don’t-shoot scenario) hereafter. The data of all 60 participants were considered for the analysis.

Every incorrect decision (i.e., false positive and false negative) was given the value “0.” In contrast, every correct decision (shooting in a shoot scenario or not shooting in a don’t-shoot scenario) was given the value “1.” By subtracting the pretest score from the posttest score, we produced a value that indicated each participant’s progress (“−1” = deteriorated, “0” = constant, “1” = improved). We then calculated non-parametric Mann–Whitney *U* tests for the decision-making progress in the shoot and don’t-shoot scenarios, respectively.

*Response Time:* This variable aimed to investigate the time of the initial motor response (firing the service pistol) after detecting the threat in shoot scenarios (hypothesis 3). We timestamped the moment in every shoot scenario when the drawn pistol was first identifiable for the participant and the moment of the first shot hitting the canvas. *Response Time* describes the time between these timestamps in milliseconds (regardless of whether the shot hit the suspect or not). To avoid distorted results in the analysis, we excluded 19 participants who did not shoot in at least one of the two shoot scenarios.

For the statistical analysis, we first calculated a repeated-measures ANOVA with the between-subject factor Group and the within-subject factor Time. We then calculated separate repeated-measures ANOVAs for each group to investigate their respective pre- to posttest development.

*First Hit:* This variable describes the time it took participants to engage the attacker with effective fire (hypothesis 4). For every shoot scenario, we calculated the time between when the attacker’s pistol was first clearly visible to the officer and the time of the first shot to hit the attacker’s torso or head. Shots outside these hit zones, which active police trainers had defined, were disregarded. We excluded 22 participants who failed to hit the target zone in at least one of the shoot scenarios.

For the statistical analysis, we first calculated a repeated-measures ANOVA with the between-subject factor Group and the within-subject factor Time. We then calculated separate repeated-measures ANOVAs for each group to investigate their respective pre- to posttest development.

*Muzzle Position:* This variable describes the time that the service pistol was held at eyesight level and pointed at the suspect before the first shot (hypothesis 5). A tactical “high ready position,” where the weapon is held slightly below eyesight level and is not yet aimed, was not considered eyesight level. We excluded the same 19 participants as in the calculation of *Response Time* since they did not shoot in at least one of the two shoot scenarios.

For the statistical analysis, we first calculated a repeated-measures ANOVA with the between-subject factor Group and the within-subject factor Time. We then calculated separate repeated-measures ANOVAs for each group to investigate their respective pre- to posttest development.

*Closed Eye(s):* This variable aimed to show whether participants kept both eyes open when using their service pistol. More specifically, it describes the time a participant had at least one eye closed right before their first shot in milliseconds (hypothesis 6). If a participant had both eyes open while aiming and firing, the value of this variable was set to “0 ms.” Again, we excluded the same 19 participants as in the calculation of *Response Time* since they did not shoot in at least one of the two shoot scenarios.

For the statistical analysis, we first calculated a repeated-measures ANOVA with the between-subject factor Group and the within-subject factor Time. We then calculated separate repeated-measures ANOVAs for each group to investigate their respective pre- to posttest development.

## Results

### Hit Factor

We had 59 evaluable data sets (intervention group = 35; control group = 24) for the *Hit Factor* analysis. The repeated-measures ANOVA with the within-subject factor Time and the between-subject factor Group revealed a significant effect with a medium effect size for the factor Time [*F*(1,57) = 8.094; *p* = 0.006; $\eta_{p}^{2}=0.124$] and a significant effect with a medium effect size for the interaction of the factors Time × Group [*F*(1,57) = 4.169; *p* = 0.046; $\eta_{p}^{2}=0.068$]. The test of between-subject effects revealed a non-significant effect with a negligible effect size for the factor Group [*F*(1,57) = 0.009; *p* = 0.924; $\eta_{p}^{2}=0.001$].

The separately calculated repeated-measures ANOVAs showed a significant main effect of the factor time with a large effect size for the control group [*F*(1,23) = 9.932; *p* = 0.004; $\eta_{p}^{2}=0.302$] and a non-significant main effect of the factor time with a small effect size for the intervention group [*F*(1,34) = 0.400; *p* = 0.531; $\eta_{p}^{2}=0.012$]. The results show that the control group significantly increased their *Hit Factor* from pre- to posttest (0.56 vs. 0.72), while the intervention group did not improve significantly (0.62 vs. 0.65).

### Decisions

**FIGURE 5:**
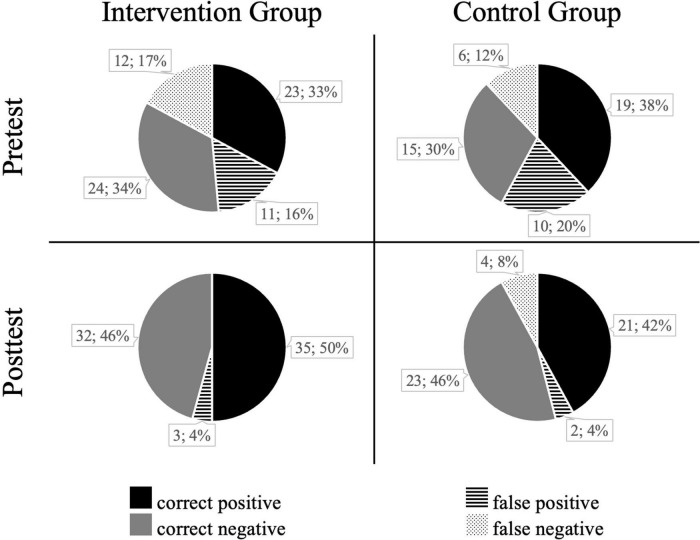
Both groups’ shoot/don’t-shoot decisions.

Figure 5 provides the number of correct positive and false negative decisions (shoot scenarios) and correct negative and false positive decisions (don’t-shoot scenarios) for the intervention and control group. Both groups started with comparable performances in the pretest (Figure 5, top), making 67–68% correct decisions (Figure 5, gray and black solid). In the posttest (Figure 5, bottom), the intervention group made 96% correct decisions (with no false negatives) while the control group made 88% correct decisions.

The statistical analyses (non-parametric Mann–Whitney *U* tests) showed that the intervention group improved their decision-making performance in the shoot scenarios (hypothesis 1) from pre- to posttest more than the control group (mean progress for intervention group = 0.34 and control group = 0.08; *U* = 393.5; *p* = 0.033). For the progress variable on decisions in don’t-shoot scenarios (hypothesis 2), the Mann–Whitney *U* test did not reveal a significant difference between the groups (mean progress for intervention group = 0.23 and control group = 0.32; *U* = 546.5; *p* = 0.435).

Table 4 provides an overview of both groups’ *Decisions*. It also shows the number of individual improvements, consistencies, and deteriorations for the shoot- and don’t-shoot scenarios, respectively.

**TABLE 4:** Overview of the decision progress from pre- to posttest in the intervention and control group (*N* = 60).

	Shoot scenarios		don’t-shoot scenarios	
	Intervention	Control	Intervention	Control
Improved (+1)	12	3	9	10
Consistent (0)	23	21	25	13
Deteriorated (−1)	0	1	1	2
Mean progress	0.34 (SD = 0.482)	0.08 (SD = 0.4)	0.23 (SD = 0.490)	0.32 (SD = 0.627)
Mann–Whitney *U*	*U* = 393.5 (*p* = 0.033)		*U* = 546.5 (*p* = 0.435)	

### Response Time

**FIGURE 6:**
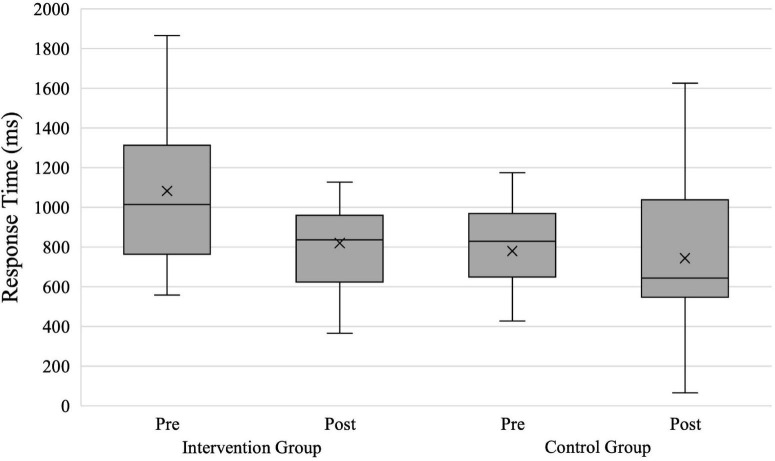
*Response Time* in shoot scenarios for both groups. X, mean marker.

For the analysis of the participants’ response times (hypothesis 3), we had 41 evaluable data sets (intervention group = 23; control group = 18). Figure 6 shows the groups’ response times in shoot scenarios. The repeated-measures ANOVA with the within-subject factor Time and the between-subject factor Group revealed a significant effect with a medium effect size for the factor Time [*F*(1,39) = 5.680; *p* = 0.022; $\eta_{p}^{2}=0.127$] and a significant effect with a medium effect size for the factor Group [*F*(1,39) = 4.791; *p* = 0.035; $\eta_{p}^{2}=0.109$]. The interaction between the factors Time × Group was non-significant with a medium effect size [*F*(1,39) = 3.248; *p* = 0.079; $\eta_{p}^{2}=0.077$].

The separately calculated repeated-measures ANOVAs showed a significant main effect of the factor time with a large effect size for the intervention group [*F*(1,22) = 9.260; *p* = 0.006; $\eta_{p}^{2}=0.296$] and a non-significant main effect of the factor time with a small effect size for the control group [*F*(1,17) = 0.167; *p* = 0.688; $\eta_{p}^{2}=0.01$]. The results confirm that the intervention group significantly reduced their response time from pre- to posttest (1,083 vs. 820 ms), while the control group did not improve significantly (780 vs. 743 ms).

### First Hit

**FIGURE 7:**
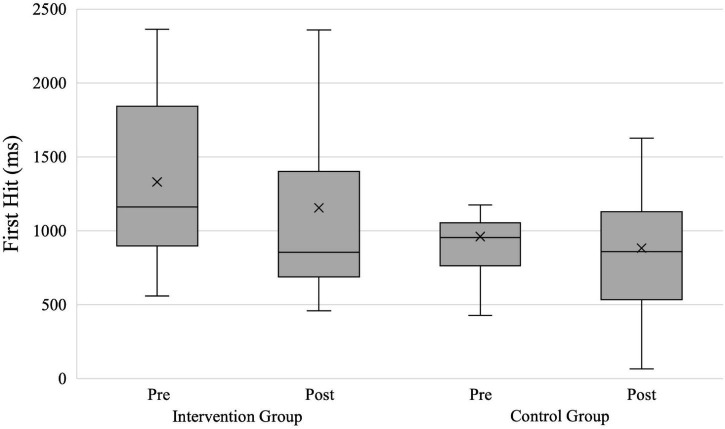
*First Hit* in shoot scenarios for both groups. X, mean marker.

For the analysis of the participants’ *First Hit* on the predefined target zone (hypothesis 4), we had 38 evaluable data sets (intervention group = 21; control group = 17). Figure 7 shows the groups’ hit times in shoot scenarios. The repeated-measures ANOVA with the within-subject factor Time and the between-subject factor Group revealed a significant effect with a medium effect size for the factor Group [*F*(1,36) = 4.607; *p* = 0.039; $\eta_{p}^{2}=0.113$] and a non-significant effect with a small effect size for the factor Time [*F*(1,36) = 0.984; *p* = 0.328; $\eta_{p}^{2}=0.027$]. The interaction between the factors Time × Group was non-significant with a negligible effect size [*F*(1,36) = 0.140; *p* = 0.710; $\eta_{p}^{2}=0.004$].

The separately calculated repeated-measures ANOVAs showed a non-significant main effect of the factor time with a small effect size for the intervention group [*F*(1,20) = 0.802; *p* = 0.381; $\eta_{p}^{2}=0.039$] and control group [*F*(1,16) = 0.276; *p* = 0.606; $\eta_{p}^{2}=0.017$], respectively. The results show that the intervention group (1,330 vs. 1,155 ms) and control group (961 vs. 882 ms) did not reduce their mean times until the first hit significantly from pre- to posttest.

### Muzzle Position

**FIGURE 8:**
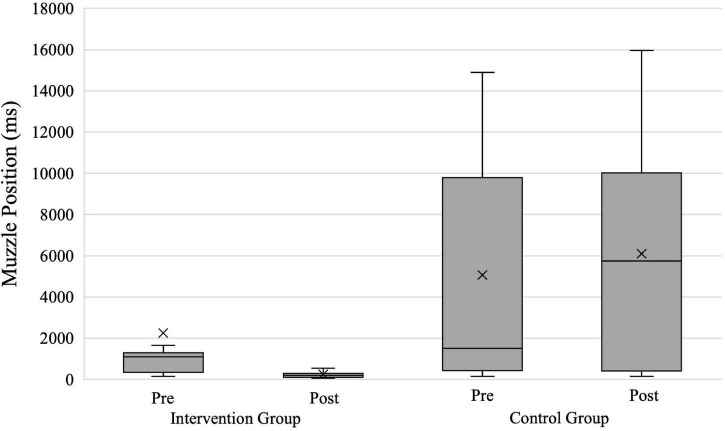
*Muzzle Position* in shoot scenarios for both groups. X, mean marker.

For the analysis of the time that participants had their weapons up to eyesight level before shooting (hypothesis 5), we had the same 41 evaluable data sets as for *Response Time* (intervention group = 23; control group = 18). Figure 8 shows both groups’ results for *Muzzle Position* in ms. The repeated-measures ANOVA with the within-subject factor Time and the between-subject factor Group revealed a significant effect with a large effect size for the factor Group [*F*(1,39) = 13.230; *p* < 0.001; $\eta_{p}^{2}=0.253$] and a non-significant effect with a small effect size for the factor Time [*F*(1,39) = 0.520; *p* = 0.475; $\eta_{p}^{2}=0.013$]. The interaction between the factors Time × Group was significant with a medium effect size [*F*(1,39) = 5.114; *p* = 0.029; $\eta_{p}^{2}=0.116$].

The separately calculated repeated-measures ANOVAs showed a significant main effect of the factor time with a large effect size for the intervention group [*F*(1,22) = 5.778; *p* = 0.025; $\eta_{p}^{2}=0.208$] and a non-significant main effect of the factor time with a small effect size for the control group [*F*(1,17) = 0.912; *p* = 0.353; $\eta_{p}^{2}=0.051$]. The results confirm that the intervention group significantly reduced the time of holding their service weapons at eyesight level before firing from pre- to posttest (2,250 vs. 247 ms), while the control group did not improve significantly (5,066 vs. 6,100 ms).

### Closed Eye(s)

**FIGURE 9:**
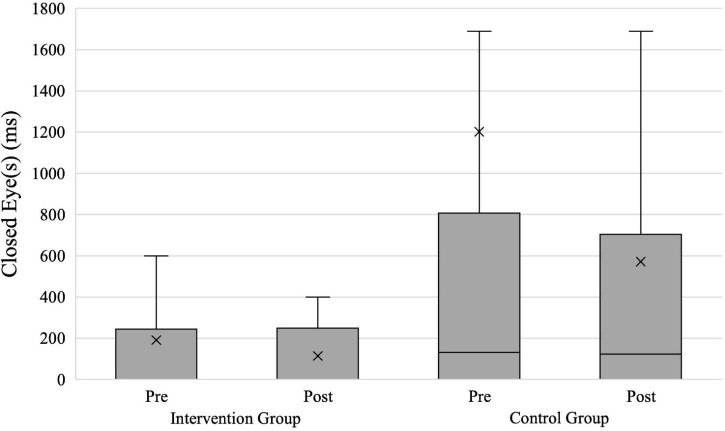
*Closed Eye(s)* in shoot scenarios for both groups. X, mean marker.

For the analysis of the time that participants had one eye closed immediate before shooting (hypothesis 6), we had the same 41 evaluable data sets as for *Response Time* (intervention group = 23; control group = 18). Figure 9 shows both groups’ results for *Closed Eye(s)* in ms. The repeated-measures ANOVA with the within-subject factor Time and the between-subject factor Group approached significance with a medium effect size for the factor Group [*F*(1,39) = 4.051; *p* = 0.051; $\eta_{p}^{2}=0.094$] and a non-significant effect with a small effect size for the factor Time [*F*(1,39) = 0.886; *p* = 0.352; $\eta_{p}^{2}=0.022$]. The interaction between the factors Time × Group was not significant and showed a small effect size [*F*(1,39) = 0.543; *p* = 0.466; $\eta_{p}^{2}=0.014$].

The separately calculated repeated-measures ANOVAs showed a non-significant main effect of the factor time with a medium effect size for the intervention group [*F*(1,22) = 2.366; *p* = 0.138; $\eta_{p}^{2}=0.097$] and a non-significant main effect of the factor time with a small effect size for the control group [*F*(1,17) = 0.550; *p* = 0.468; $\eta_{p}^{2}=0.031$]. The results show that neither group significantly reduced their *Closed Eye(s)* time from pretest to posttest (intervention group = 190 vs. 113 ms; control group = 1,202 vs. 571 ms).

## Discussion

The present study took place in an indoor firing range using live ammunition. The aim was to examine police cadets’ shoot/don’t-shoot performances in video scenarios before and after two different types of firearms training. The control group received traditional firearms training, while the intervention group received specifically developed firearms training based on state-of-the-art empirical findings in cognitive psychology. The study investigated whether training focused on tactical gaze control and visual attention tactics improves decision-making performance and (tactical) response preparation and execution more than traditional firearms training. Both the control group and intervention group completed a pretest before—and a posttest after—their respective training. Throughout the study, it was notable that the use of live ammunition offered a considerable level of realism and caused the participants to behave more cautiously and alertly than they typically do when they use non-lethal training equipment.

The results of the manipulation check *Hit Factor* show that only the control group improved their performance from pre- to posttest in this static and abstract task, while the performance of the intervention group stayed consistent. This outcome is reasonable since the control training aimed to improve the traditional aspects of firearms proficiency: hitting a geometrical shape in the least amount of time possible. On the other hand, the intervention training focused on training participants to make conscious shoot decisions based on threat assessments. This illustrates that one cannot assume that decision-making and threat-assessment training will automatically improve traditional marksmanship performance.

As long as police academies base their evaluations of officers’ firearms proficiency on “how many predefined targets were hit in what time?” traditional training will seem to generate the best shooters. However, it must be considered that these tasks are highly abstract and do not resemble real-life scenarios. Training officers to hit targets 10 mm closer to the bullseye or reducing response time by 10 ms must not be considered more important than training officers to make correct shoot decisions in the first place.

This study’s intervention training aimed to improve police officers’ decision-making performances in shoot/don’t-shoot scenarios by implementing theory-based aspects of tactical gaze control, visual attention tactics, and situational awareness. Participants were encouraged to prioritize the underlying processes of making a shoot decision (visual perception, threat assessment, and conscious decision-making) over sports-like factors such as shooting as fast and accurately as possible.

Concerning our hypotheses, we found that the intervention group improved their number of correct decisions to shoot in shoot scenarios to a greater extent than the control group (hypothesis 1): while 34% of the intervention group improved their pre- to posttest performance, only 12% of the control group improved. Furthermore, one control group participant even scored lower after the training than before.

We also assumed that the intervention group would shoot faster (hypothesis 3) in shoot scenarios than the control group. Our results showed that the intervention group reduced their *Response Time* significantly by 263 ms, while the control group did not reduce it to a degree of statistical significance. At this point, the groups’ differing baseline performances must be taken into consideration. The intervention group started with a higher mean response time, which may have amplified the observed effect.

Moreover, we hypothesized that the intervention group would bring their gun up to eyesight level later (hypothesis 5) before shooting than the control group. Our analysis revealed that only the intervention group reduced their *Muzzle Position* significantly (by 2,003 ms). This leads us to conclude that the intervention group participants understood the value of keeping a clear line of sight toward the suspect. Therefore, they may have waited longer until they raised their weapon as this tends to block the vision of the suspect’s hand and hip region. This result matches the intervention’s training goals and aligns with findings suggesting that a lower muzzle position improves shoot/don’t-shoot decision-making (Taylor, 2020).

Furthermore, we hypothesized that the intervention group would make the correct decision not to shoot in don’t-shoot scenarios more often than the control group (hypothesis 2). However, our analysis of *Decisions* did not reveal a significant difference in both groups’ decision-making progresses in don’t-shoot scenarios. Therefore, hypothesis 2 was not confirmed. However, both groups improved their decision-making performance in don’t-shoot scenarios from pre- to posttest. This indicates an unspecific training effect, potentially caused by the pretest scenarios.

We also assumed that the intervention group would hit the attacker faster than the control group (hypothesis 4). However, neither group reduced their *First Hit* significantly from pre- to posttest, thus not confirming hypothesis 4. We suspect that this can be explained by the participants’ still rather basic levels of firearms proficiency. Thus, even though the intervention group may have identified threats quicker and decided to shoot faster, their underdeveloped skill levels may have prevented them from reliably hitting the attacker with their fast first shots. This could be explained by a speed–accuracy tradeoff or the assumption that the intervention group spent more mental resources on active vision and verbal communication.

For *Closed Eye(s)*, our analysis showed a tendency that the intervention group keep both eyes open longer right before shooting than the control group. This observed tendency aligns with hypothesis 6 and matches the intervention’s training goals.

Considering the overall results, it is noteworthy that although the intervention training had a noticeable positive impact on decision-making performance in shoot scenarios, performance in don’t-shoot scenarios did not differ by training type. Both groups improved their decision-making performance in don’t-shoot scenarios from pre- to posttest. The 90 min of intervention training were apparently enough to improve the participants’ ability to detect and identify the firearms drawn by the attacker. However, the 90 min of intervention training did not affect the participants’ ability to distinguish harmless from dangerous objects or suppress the shooting motion (inhibition) under stress. Another point worth considering is that the intervention group were encouraged to use verbal communication tactics when interacting with a potentially armed suspect. In contrast, the control group’s traditional firearms training did not include verbal aspects. Although all participants have had previous training on basic verbal communication tactics, it became apparent that the workload was already very high in the scenarios and the use of verbal communication tactics became even more demanding. Therefore, the workload of the intervention group – who were reminded of the importance of verbal communication tactics in their intervention training just before the posttest – was probably higher when addressing the suspect correctly than the control group’s, who in some cases did not communicate verbally at all. Just reacting is less challenging than reacting while actively communicating.

### Limitations and Future Studies

The current study was conducted at a police academy using live ammunition, official equipment, and a very selective sample: police cadets. Therefore, the results of this job-related intervention are highly targeted and do not necessarily translate to a more general population. On a methodological level, we faced the limitation that we could not randomly assign the participants to the groups as we had to work with intact classes. This quasi-experimental design could have generated distortions caused by different skill levels between the groups. However, we expected these effects to level out due to the number of groups.

Due to the previously described technical issues, nine datasets of the control group were not recorded during the posttest. This left us with uneven group sizes (intervention group = 35; control group = 25), possibly causing a loss of power in the statistical analysis.

During the study, it became apparent that the learning effects from the pretest were considerable. A third group omitting the pretest could have been helpful to distinguish learning effects caused by the pretest from effects linked to the actual training. Another valuable addition for future studies would be introducing a retention test. Including a retention test—for example, 4 weeks after the posttest—would make it possible to evaluate the sustainability of the intervention.

Future studies could also focus on more experienced officers and investigate whether the participants’ overall levels of firearms proficiency correlate with their ability to benefit from theory-based shoot/don’t-shoot decision training. Another point worth looking into deeper is the relationship of muzzle position, response time, and shoot/don’t-shoot decision-making. For example, finding practical ways to benefit from a lower muzzle position while keeping the extra response time to a minimum could lead to promising new training approaches. Furthermore, we believe that signal detection theory offers promising opportunities to investigate the underlying processes of shoot/don’t-shoot decision-making in law enforcement.

### Conclusion and Practical Implementation

During the pre- and posttest, it was noticeable that using service weapons with live ammunition added a considerable degree of realism and stress to the experiment. Even participants who were generally comfortable with weapon handling and basic firearms tactics showed apparent signs of stress-related limitations during the video scenarios. We assume that most participants would be less stressed in experiments using non-lethal training equipment like marking rounds (special weapons shooting soft colored projectiles that leave visible marks) or laser weapons with simulated recoil. However, non-lethal training equipment can provide other aspects of realism, like face-to-face scenarios with actors and complex 360° scenarios in real-world locations. Therefore, we recommend a balanced mix of theory-based training both with live ammunition and non-lethal alternatives.

Another crucial aspect of a professional police force is the trainers’ and trainees’ awareness that regular practice is vital. Fundamental “dry drills” without the use of live ammunition (for example, drawing, reloading, holstering, and clearing malfunctions) can be practiced virtually anywhere and anytime to automatize sequences of movements. Just understanding tactical concepts is not enough to be prepared for highly complex and stressful real-life situations. Only a combination of theory, well-designed basic training, and further regular training provides law enforcement personnel with the necessary tools to handle potentially deadly situations with a maximum degree of professionalism. Officers must not only train to reduce the risk of losing their own life but also to reduce the risk of taking an innocent life.

The 90 min of theory-based firearms training showed considerable positive effects on participants’ shoot/don’t-shoot performance and weapon handling. These effects, however, seem to have been hampered by the still basic level of the participants’ firearms proficiency and the brief nature of this one-time intervention. We surmise that regular training of this sort – not only with cadets but also with experienced officers – could further increase the observed effects. In particular, the shoot/don’t-shoot scenarios seemed to have had a considerable “Aha effect” on the participants, which led them to better understand the nature of the split-second decisions they may have to make while performing their operational duties.

Law enforcement agencies need to further focus their firearms training on decision-making and tactical perception. Police officers, who shoot fast and accurately, are only good shooters if they base their shoot decisions on valid threat assessments. Making the correct decision to shoot or not shoot under stress and with limited time is an incredibly demanding task. Therefore, law enforcement agencies should be obliged to regularly renew their officers’ firearms competency licenses based on well-designed tests. The tests for this renewal process must comprise not only aiming tasks but also perception-based shoot/don’t-shoot tasks. Law enforcement firearms proficiency must not be confused with proficiency in competitive shooting sports.

Police officers should understand the tactical importance of a potential attacker’s hands and hip region (cf., Heusler and Sutter, 2020a). In almost all cases, an attacker will have to use their hands to pose an immediate deadly threat. Although a suspect’s face is a salient stimulus and may reveal some observable emotions, neglecting tactically more important regions could potentially be fatal. Faces do not operate firearms and knives – hands do!

In the current study, it was notable that some participants lowered their weapons immediately after the first shot and relaxed noticeably. However, police officers must understand that a single shot at an armed attacker might not instantly stop the immediate threat, even if the shot is well placed. Therefore, shoot/don’t-shoot training should also train officers to reassess critical situations continually. Proper reassessment of an ongoing attack may result in the realization that further actions must be taken to stop the imminent threat.

The scientific investigation of police de-escalation tactics and their effectiveness is a relatively young field of study and has produced somewhat inconclusive results so far (Oliva et al., 2010; Todak, 2017; Todak and James, 2018; Todak and White, 2019; Bennell et al., 2020; Engel et al., 2020). However, as studies become more mature and findings accumulate, this field of research may provide valuable insights into equipping officers with more tools to find peaceful solutions for potentially deadly encounters – especially when combined with situational awareness, gaze control, and tactical attention strategies.

Li et al. (2020) found that non-stress law enforcement training is associated with significant reductions in police officers’ use of deadly force. This approach may facilitate a citizen-oriented “guardian-mindset” as opposed to a militarized “warrior-mindset” in law enforcement (cf., Stoughton, 2016). Other research, however, suggests that training under elevated levels of anxiety is vital for police officers to perform in real-life threat scenarios (Oudejans, 2008; Nieuwenhuys and Oudejans, 2011; Liu et al., 2018). Instead of focusing too much on creating either guardians or warriors, we strongly advise giving officers the necessary tools (material, education, and training) to fulfill both roles. We believe that situational awareness and mental preparedness are the keys to a citizen-oriented police force that is also capable of withstanding robust confrontations.

A step toward modernizing police use-of-force training could be introducing a theory-based multidimensional model of law enforcement firearms competency. Traditional and outdated ways of evaluating firearms proficiency (like only relying on the previously described Hit Factor) should be complemented with aspects such as perception, decision-making, weapon handling, verbal communication, etc. This could substantially improve individual law enforcement agencies’ abilities to evaluate their personnel’s firearms proficiencies. Shooting quickly and accurately on predefined geometrical shapes is not as crucial for police officers as basing shoot decisions on valid threat assessments.

## Data Availability Statement

The raw data supporting the conclusions of this article will be made available by the authors, without undue reservation.

## Ethics Statement

The studies involving human participants were reviewed and approved by the Ethical Review Committee of the German Police University. The patients/participants provided their written informed consent to participate in this study. Written informed consent was obtained from the individual(s) for the publication of any potentially identifiable images or data included in this article.

## Author Contributions

Both authors listed have made a substantial, direct, and intellectual contribution to the work, and approved it for publication.

## Conflict of Interest

The authors declare that the research was conducted in the absence of any commercial or financial relationships that could be construed as a potential conflict of interest.

## Publisher’s Note

All claims expressed in this article are solely those of the authors and do not necessarily represent those of their affiliated organizations, or those of the publisher, the editors and the reviewers. Any product that may be evaluated in this article, or claim that may be made by its manufacturer, is not guaranteed or endorsed by the publisher.
